# Soft Hardware, Flowing Software: Reconfigurable Microfluidics for Adaptable Chemical Computation

**DOI:** 10.1002/adma.73669

**Published:** 2026-06-10

**Authors:** Piet J. M. Swinkels, Brigitta Dúzs, Oliver Skarsetz, Kohei Nishiyama, Andreas Walther

**Affiliations:** ^1^ Life‐Like Materials and Systems Department of Chemistry University of Mainz Mainz Germany

**Keywords:** 3D printing, chemical reaction network theory, computation, computer science, DNA nanotechnology, logic gate, microfluidics, reservoir computing, smart hydrogels, software

## Abstract

Chemical and physical computing systems promise information processing in performance regimes inaccessible to conventional electronics. However, they are typically constrained by static hardware architectures that limit adaptability and computational richness. Here, we introduce a reconfigurable microfluidic platform where soft hydrogel structures are 3D‐printed and erased in situ to dynamically reshape the physical environment in which chemical computation occurs. By treating microfluidic geometry as an active, programmable element rather than a passive container, we demonstrate hardware‐reconfigurable control over chemical information processing. We demonstrate switchable Deoxyribonucleic acid (DNA) logic gates that alternate between AND and OR functionality without modifying the underlying reaction network, decoupling logic function from molecular composition. Extending this to a non‐equilibrium chemical reaction network in the form of a feedback‐controlled pH oscillator, we demonstrate that printed structures steer reaction kinetics and spatial pattern formation, giving rise to geometry‐dependent spatiotemporal states. Leveraging these dynamics, we implement a physical reservoir computer in which reconfigurable microfluidic hardware enables the realization of diverse nonlinear functions through simple linear readout. Our work establishes reconfigurable soft microfluidic hardware as a control layer for chemical computation, highlighting how adaptable physical environments actively expand the computational state space of chemical software.

## Graphical abstract

A reconfigurable microfluidic platform based on soft, photo‐printable, and chemically erasable hydrogel structures printed and erased in situ is used to control flow routing, mixing, chemical patterning, and even chemical computing. Using hardware to control chemical computations decouples logic function from molecular composition, demonstrated via switchable DNA logic gates and a reservoir computer based on a pH oscillator.

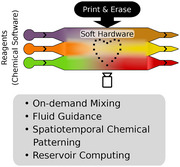

## 1 Introduction

Physical and chemical computing aim to realize information processing by harnessing intrinsic physical and chemical dynamics rather than relying on silicon‐based electronic circuits. They promise capabilities that are difficult to achieve with conventional electronics, including massive parallelism, scalability, and neuromorphic signal processing [1, 2, 3, 4]. These advantages are especially compelling in environments where conventional electronics fall short: wet, chemically complex, and energy‐poor settings like biological tissues. While early work has primarily explored fundamental questions of computation and intelligence, experimental implementations are increasingly demonstrating how these principles can be leveraged for functional tasks in, e.g., sensors, classification, and soft robotics [5, 6, 7, 8, 9].

Such computing can be implemented through two complementary approaches: binary logic, analogous to conventional computers, or network‐based architectures inspired by biological systems in which neurons or biomolecules collectively process information. While binary systems are comparatively simple to implement and interpret, network‐based approaches promise advantages like massive parallelism, enhanced scalability, and robustness to noise. The components performing the chemical calculations are typically referred to as *software*: programmed to perform specific computational tasks, they act analogously to traditional computer software. In terms of software for chemical computing, the high specificity of DNA reactions has made them well suited for binary computations [10, 11, 12, 13]. For instance, a 1‐bit full adder – one of the fundamental building blocks of modern Central Processing Unit (CPUs) – has been implemented using only 13 DNA strands [14]. In contrast, chemical reaction networks (CRNs) [15] with higher complexity and often involving non‐linear feedback lend themselves naturally to network‐based computation [16, 17, 18, 19, 20], as demonstrated by reservoir computing realized in the formose reaction [21]. Autocatalytic CRNs, famous for producing complex Turing patterns, can also be used to perform binary and networked computational tasks [22, 23, 24]. However, a persistent limitation with many chemical computing systems is their typically ‘one‐shot’ nature: only one calculation can be performed before the system must be reset, typically requiring replenishment of starting materials [25, 26, 27, 28].

This constraint severely limits scalability and practical applicability beyond the lab. A potential solution is to operate calculations under continuous microfluidic flow, allowing calculations to take place inside a moving ‘local environment’ that is advected along the stream [29, 30]. This allows the input to be modulated to perform calculations continuously. Furthermore, appropriate systems can yield a memory in the obtained neural network, substantially enhancing computational power [21, 31, 32]. Moreover, beyond enabling continuous operation, microfluidic environments can themselves add additional control mechanisms to expand computational capacity [33]. Indeed, purely microfluidics‐based logic computations have been implemented [34], highlighting the potential of flow geometry to influence fluidic computation. However, most existing approaches rely on fixed, highly specialized chip designs that require complex fabrication workflows to realize different computational functions [35]. Consequently, strategies are needed that enable rapid, reliable, and reversible adaptation of microfluidic architectures – on demand [36, 37, 38] and ideally without changing the chip.

Herein, we introduce reconfigurable microfluidic systems built from photo‐printable and chemically erasable hydrogel inks that serve as customizable obstacles, mixers, and navigation routes for multi‐input/multi‐output microfluidic chips and demonstrate their use to create and enhance the capabilities of chemical computations. We showcase the operation of this ‘soft hardware’ for DNA‐based logic gates whose operation can be changed by reconfiguring chip flow, and for simple chemical computers based on an autocatalytic CRN (a pH oscillator), which sense objects of the printed soft hardware in the microfluidic chip.

## 2 Results

### 2.1 Reconfigurable Microfluidics

**FIGURE 1:**
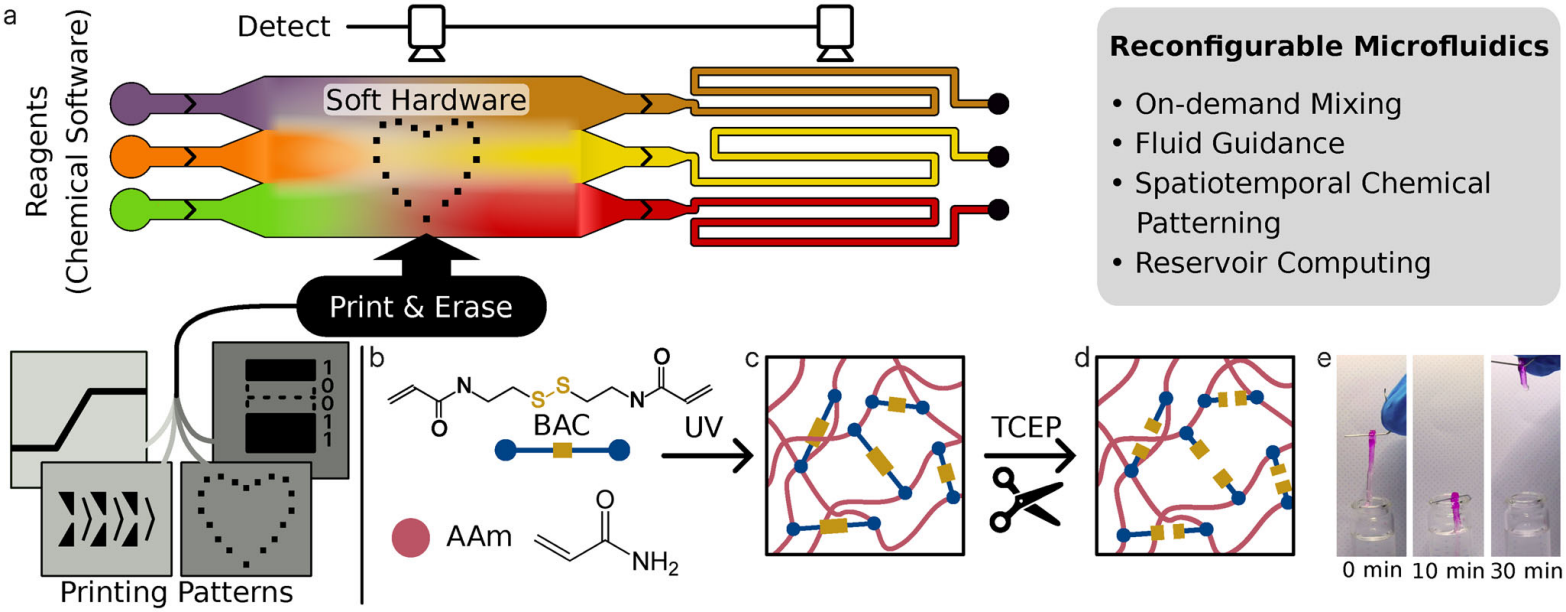
Chemical software is guided by reconfigurable soft hardware. (a) Overview of how we use reconfigurable microfluidics as dynamic microfluidic hardware, leading to various rich behaviors when combined with selected chemical software. (b‐d) The reconfigurable microfluidic system is based on printing and erasing of hydrogel patterns formed by acrylamide monomer (AAm, red) and *N, N'*‐bis(acryloyl)cystamine (BAC, blue/yellow) cross‐linker upon photo‐polymerization. Addition of a reducing agent (like TCEP) cuts the cross‐links, leading to hydrogel dissolution. (e) Example of a hydrogel dissolving in a vial containing a 50 mm TCEP solution.

Our reconfigurable microfluidic system is based on a soft rewritable hardware that can be photo‐printed inside a glass microfluidic chip, chemically erased, and reprinted. Figure 1 summarizes the entire concept, its capabilities, and the hydrogel resin developed. The hydrogel formulation is composed of a monomer mixture of commercial acrylamide monomers (AAm) and *N, N'*‐Bis(acryloyl)cystamine (BAC) cross‐linkers that can undergo radical polymerization into solid hydrogels. BAC contains a disulfide bond that can be cleaved under reducing conditions, leading to dissolution of the hydrogel [39]. Figure 1b–e depict the chemistry and the process at ambient conditions for a hydrogel photo‐polymerized at 12.6 wt.% (AAm/BAC = 150/1 mol/mol) with 0.3 wt.% LAP (LAP = Lithium‐Phenyl‐2,4,6‐trimethylbenzoylphosphinate, photo‐initiator) and subsequently incubated in a 50 mM TCEP (TCEP = Tris(2‐carboxyethyl)phosphine hydrochloride). Dissolution of the hydrogel strut with a diameter of 1.5 mm occurs within less than 30 min. Other reducing agents have similar effects (Note S1).

**FIGURE 2:**
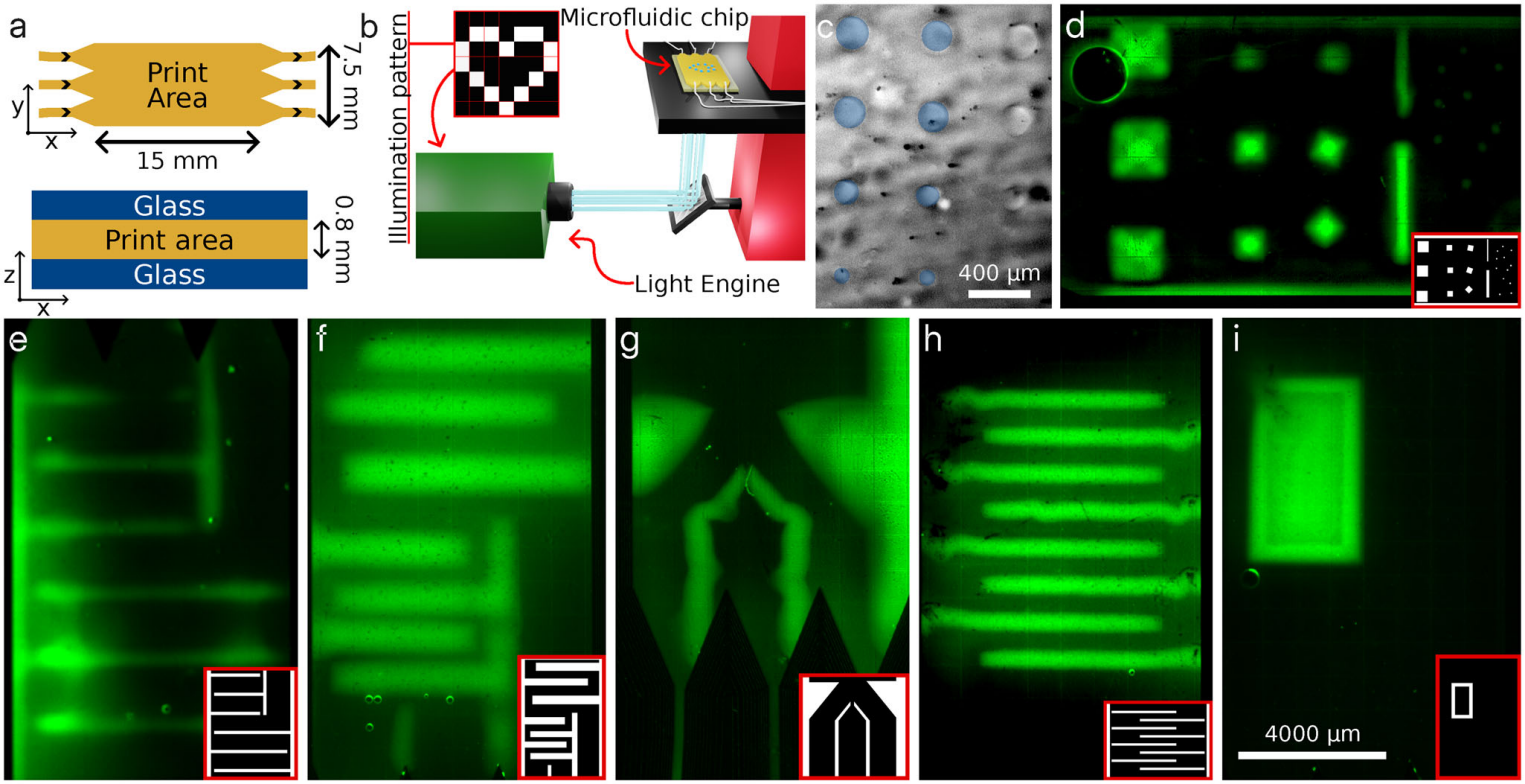
Printing reconfigurable microfluidic features using erasable hydrogel inks inside glass microfluidic chips. (a,b) Scheme of our printing setup and microfluidic chip. A UV‐light pattern (385 nm) is projected into the chip using a light engine (2560 × 1600 resolution, 6 µm/pixel). (c) Brightfield microscopy image of printed dots down to 50 µm in diameter in a microfluidic chip (blue false color for the two left rows), representing the approximate lower limit of our printing resolution with the reconfigurable resin. (d‐h) Different printing patterns as examples with insets showing the DMD exposure mask. All patterns were printed consecutively in the same chip, highlighting the efficient print‐erase cycle. (i) Printing of a rectangular container confines liquid resin inside the container, confirming printing across the entire thickness. The scale bar in panel i applies to panels d‐i. Fluorescent wide field imaging was performed after addition of ∼0.02 mol% of Rhodamine B‐containing comonomer.

We apply this reversible hydrogel as a reconfigurable microfluidic system by 3D‐printing inside a glass microfluidic chip (Figures 1a and 2) [40]. The chip chamber has dimensions of 7.5 mm × 15 mm with a height of 0.8 mm and features 3 entries and 3 exits, which are all fixed. We print erasable and thus reconfigurable patterns (obstacles, mixers, flow navigators) inside this microfluidic chip using an optical printing setup developed in‐house, consisting of a light engine (a digital micro‐mirror device (DMD)‐based image projector), that projects UV‐light into the microfluidic chip to locally photo‐polymerize the displayed pattern (Figure 2b). The displayed image polymerizes hydrogels as microfluidic components with resolution down to approx. 50 µm (Figure 2c). Since the light engine allows flexible input images, this effectively yields a system allowing to print arbitrarily complex 2D microfluidic circuitry within the glass chips.

Six representative examples demonstrate the flexibility regarding printing (Figure 2d–i). All these prints were made in the same chip following print‐image‐erase cycles (see Methods). Erasing is achieved by flowing a 50 mm TCEP solution through the chip, after which prints break down in between 10 and 30 min, depending on the size of the print. There is no clear upper limit to the number of print‐erase cycles that can be performed in a chip, as surface modifications on the glass are not necessary (Note S2). Importantly, to function as efficient microfluidic obstacles, the printed patterns need to go through the entire thickness (0.8 mm) of the chip (Note S3). Figure 2i demonstrates one example in which a printed container effectively confines the liquid fluorescent resin within, whereas other areas of the chip are cleaned by a flushing step.

**FIGURE 3:**
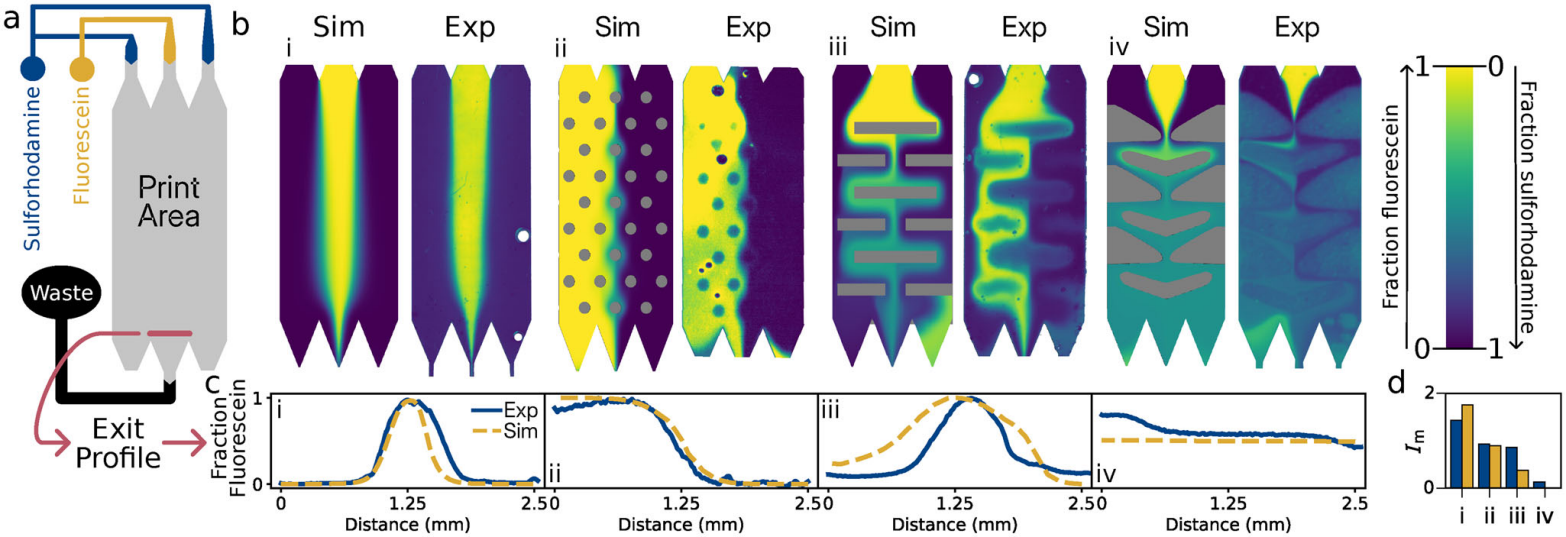
Mixing performance of reconfigurable microfluidic mixers. (a) Schematic of setup and detection point. (b) Mixing of sulforhodamine 101 and fluorescein using different mixers (i‐iv). Each panel contains the simulated (left) and experimental (right) results. (c) Normalized fluorescence intensity profiles across the exit line marked in red in panel a, of prints (i‐iv) comparing simulation (yellow dashed) and experiment (blue solid). (d) The mixing index *I*_m_ of the four different printed patterns (i‐iv), with both simulations (yellow) and experiments (blue) indicated.

To demonstrate that the functionality of the same microfluidic chip can be altered by printing different structures, we perform a range of experiments with different mixers. Mixing is a classical challenge in microfluidics because flow is laminar, and macroscopic mixers that base their function on turbulence are ineffective. Instead, (passive) microfluidic mixers work by enhancing molecular diffusion and generating chaotic advection [41]. We monitor the mixing of two aqueous dye streams, sulforhodamine 101 and fluorescein, using fluorescence microscopy (Figure 3a). We use a flow rate of 0.5 mL/h, where printed structures remain stable (Figure S1). Tandem finite element simulations (COMSOL) complement the picture (see Methods S1 for details). Figure 3b displays mixing behavior for different patterns, and Figure 3c compares the exit profiles of each chip to quantify the degree of mixing via the so‐called Mixing Index (*I*_m_), plotted in Figure 3d. *I*_m_ is defined as the ratio of the standard deviation to the mean intensity (e.g., the coefficient of variation) along the exit channel: *I*_m_ = σ/µ, where a mixing index of 0 indicates perfect mixing.

Mixing is minor for an empty chip (Figure 3b(i)), because the flow is laminar and mixing only occurs through diffusion. Hexagonal pillar patterns inside the chip (Figure 3b(ii)) enhance mixing slightly, but the streams are still largely separated. The *I*_m_ drops to ca. 0.9 compared to over 1 in the unprinted variant. Using bars to create a maze‐like pattern in Figure 3b(iii) yields a similar improvement, resulting in better mixing compared to the no‐print case, with *I*_m_ < 1. Finally, we used the so‐called chamber‐wedges pattern for best mixing (Figure 3b(iv)), resulting in a near‐perfect mixing of the streams in simulations, and highly efficient mixing in actuality (*I*_m_ is 0.01 and 0.13 respectively), matching literature values [42]. The slightly inferior experimental mixing stems from a slight misalignment of the mixer with the chip, which is challenging to fully eliminate experimentally.

These demonstrations underscore the easy‐to‐use and flexible nature of our developed system to achieve reconfigurable microfluidics. Furthermore, by combining programmable microfluidic pumps and an in situ imaging station, our system allows for easy automated experiments, with pre‐programmed cycles of printing, experiments, and resetting of the printed structures.

### 2.2 DNA Computation

**FIGURE 4:**
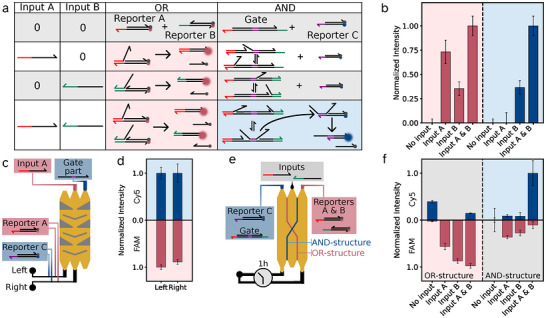
Switching between DNA‐based logic gates based on printed structures that guide fluid flows. (a) Truth table and reactions of the DNA‐based OR and AND logic gates. (b) Bulk performance of the OR (FAM signal, red) and AND (Cy5 signal, blue) gates. (c) DNA‐mixer layout: *Input A* enters left, *Gate part* enters right, are mixed by the printed structures, and detected using *Reporter A* & *C* at the chip exit. (d) Bar plot shows near‐perfect mixing by similar intensities of activating both *Reporters A & C* in both exit channels. (e) Switchable DNA logic gate layout: *Reporter C* and *Gate* (AND) enter left*, Reporters A & B* enter right (OR), and *Inputs A & B* enter in the center. Two exits collect the output with a delay of ca. 1 h to allow for reactions to take place. (f) Results as bar diagrams: the OR‐structure yields OR behavior (bottom left), the AND‐structure yields AND behavior (top right). Non‐fitting reporters (top left, OR; bottom right, AND) are only activated to a minor extent. Bars and standard deviations are from four experiments.

We will next demonstrate the application of reconfigurable microfluidic systems for programming simple microfluidics‐based chemical computations. We start by looking at DNA cascades that perform either an AND or OR logic gate through strand‐displacement reactions [14] (Figure 4a). In both cases, there are two single‐stranded DNA (ssDNA) inputs: *Input A* and *Input B*. In the OR‐gate, the presence of either of these molecules results in a fluorescent signal, while the absence does not. In the AND gate, the presence of either *Input* alone does not generate fluorescence, only if both *Inputs* are present, a fluorescent signal is generated (sequences in Table S1). The OR gate contains two double‐stranded DNA (dsDNA) reporters, each labelled with a fluorophore and a quencher*. Input A* displaces the quencher‐strand on *Reporter A*, and *Input B* does the same on *Reporter B*; both events activate fluorescence (FAM). The AND gate contains a *Gate* duplex and a *Reporter C* duplex. Either input alone binds the *Gate* without triggering fluorescence. When both inputs bind, they release a strand from the *Gate* that subsequently displaces the quencher strand on *Reporter C*, generating fluorescence (Cy5). Bulk experiments in a plate reader confirm the expected behavior (Figure 4b). The OR gate (red) shows a low FAM signal in the absence of inputs and is high when either of the inputs is present. The AND gate (blue) yields a low Cy5 signal in the absence of inputs, and when only one input is present. Once both inputs are added to the AND gate, the fluorescent signal is high.

Before implementing these DNA‐based logic gates with our reconfigurable microfluidic system, we first demonstrate that the printed structures can guide and mix DNA strands through our chips without interference with the hydrogel resin (Figure 4c). Two ssDNAs, *Input A* and one strand of the *Gate*, enter the chip from separate inlets at 1 mL/h (center and right). At the two chip outlets (left and center), we add *Reporter A* and *Reporter C* that light up in the presence of these two input strands, respectively. Measuring both reporter fluorophores at the exits with a printed chamber‐wedges pattern (a good mixer, as discussed above) reveals approximately equal *Reporter A* and *Reporter C* signals at both outlets (Figure 4d). This confirms efficient mixing of ssDNA entering the chip with our on‐demand printed structures and exemplifies our downstream detection. We further confirm DNA does not get trapped in printed structures (Figure S2).

Having established the basic function of the chip and the DNA system, we next consider a design implementation wherein the reconfigurable hydrogel pattern acts as a fluid navigator to either select the AND or the OR operation. For this, we need three input channels: *Gate* and *Reporter* on the left (AND side), *Input A* & *B* in the center, and *Reporter A* & *B* on the right (OR side), all flown in at 1 mL/h. The two outlets of the chip are kept separate for 1 h to ensure completion of any reactions before being mixed for fluorescence read‐out. By printing different walls to create two programmable paths in the chamber (red line and blue line), we can dynamically select what logic gate is active (Figure 4e). The AND‐structure (blue) brings *Gate, Reporter C*, and *Inputs* together, while the OR‐structure (red) brings *Reporters A & B* and *Inputs* together.

After running all four combinations of different inputs (00, 10, 01, 11) with the OR‐structure, we reset the chip, print the AND‐structure, and repeat. Figure 4f displays the FAM and Cy5 fluorescence signal of these eight cases. When the OR structure is printed, the FAM signal is positive when either or both inputs are present, but zero when neither is present. The Cy5 signal (corresponding to the AND gate detection), meanwhile, is always low. In contrast, for the printed AND structure, the Cy5 signal is only positive when both inputs are present, and the FAM signal is correspondingly low. Some FAM activation is visible when only one input is present, because the input strands are only transiently scavenged by the *Gate* and can over time, activate *Reporter A & B*. The observation of those signals is due to the fact that we unite both exit streams at the end for simultaneous detection.

This adaptable AND‐OR logic gate patterning shows that our reconfigurable microfluidics platform can act as a simple logic gate that can change its function on the fly, without adding additional DNA strands. It solves two major problems simultaneously: (1) it acts in‐flow, meaning that reactions are not one‐shot, and (2) it allows for additional computational complexity with the same number of reagents. The proof‐of‐principle system we present here only switches between two logic gates, but it opens the door to more complex switching, with more advanced chip designs, engineered CRNs, and, potentially, cascaded microfluidic chips allowing for significantly more complex logic systems.

### 2.3 pH Oscillator: From Standing Waves to Reservoir Computing

**FIGURE 5:**
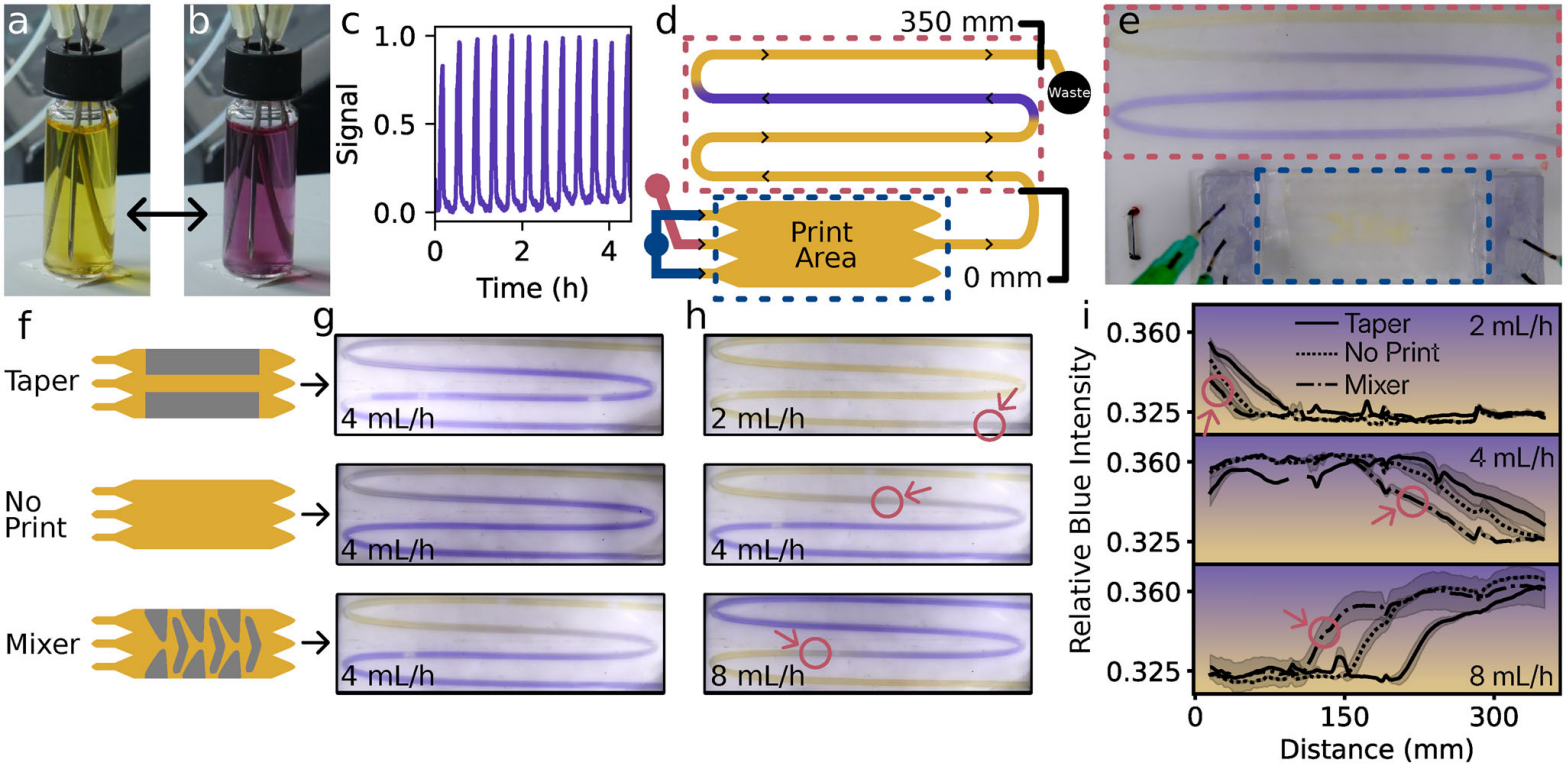
pH oscillator on a chip with reconfigurable soft hardware. (a,b) The thiosulfate‐peroxide CRN switches between low pH (yellow) and high pH (purple). (c) pH oscillations in a CSTR tracked via the normalized blue signal in RGB code. Purple pulses appear every approx. 30 min. (d,e) Schematic and picture (at 4 mL/h) of the setup for the CRN flowing through the microfluidic chip: The CRN enters unmixed into the chip from two reservoirs. The CRN flows through the printing area into a single exit channel into downstream tubing, which ends in a waste vial. A camera records color changes. (f) Used prints in the chip. (g) Steady‐state snapshots for purple bands formed at different distances depending on the print used. (h) Steady‐state snapshots for purple bands formed at different distances as a function of flow rate, here showing the ‘Mixer’ print. (i) Plot of the relative blue intensity along the length of the downstream tubing for different patterns and flow rates. The shaded band indicates the standard deviation over time. The circles help to correlate to the snapshots.

We now move beyond logic gates and establish a microfluidic system that can do simple computational work based on a CRN modulated by the objects printed inside the microfluidic chip. We use a CRN based on hydrogen peroxide, thiosulfate, and Cu(II) [43], which under the right conditions, oscillates between two states with low (approx. 3) and high pH (approx. 8.5). Bromocresol purple can efficiently visualize these states, yellow at low and purple at high pH (Figure 5a,b). Video S1 provides a demonstration within a small continuously stirred tank reactor (CSTR). When the flow rate (and thus residence time) of the CSTR is set correctly (see Methods), it oscillates between yellow and purple with a period of ca. 30 min (Figure 5c, Video S1). [Correction added on June 22, 2026, after first online publication: Textual Error in the header of section 2.3 has been corrected.]

Since time correlates to space in flow, we hypothesized that a standing wave can be produced whose position (time/space) depends on the flow and mixing conditions within a microfluidic chip. The output wave is thus a computational result of the input pattern, the degree of mixing, and the flow rate. We set up the system by flowing separate streams of hydrogen peroxide in the center (red) and two outer streams with all other components, including the indicator (yellow) into the microfluidic chip. The exit is through a single channel, connected to long tubing before entering a waste vial (Figure 5d,e). A camera records color changes along this tubing (Videos S2 and S3; note: ca. 95 mm are not captured from the chip exit to the meandering tubing due to the experimental setup). At 4 mL/h, we indeed observe a stable purple band in the tubing (Figure 5e). This band forms from yellow‐to‐purple at the edge of our field of view, just as it exits the chip, and shows a purple‐to‐yellow transition further downstream, indicative of the ongoing reaction and pH switches. The band is stable over time, although short‐term oscillations are observed due to the intrinsic dynamics of the system (Figure S3).

We next investigate the behavior as a function of flow rate and microfluidic architecture. We confirmed the hydrogel is stable inside the CRN (Figure S4), and printed three architectures: (1) no print, (2) an efficient mixer (the chamber‐wedges pattern), and (3) a taper pattern that discourages mixing by taking up volume (Figure 5f). At a given flow rate, the printed structures consistently influence the location of the standing wave. In the presence of the mixer, the standing wave stabilizes earlier than for the no‐print case, which stabilizes earlier than the taper print (Figure 5g). For a fixed print (Figure 5h), higher flow rates push the location of the standing wave further downstream. At 2 mL/h, the purple band hardly enters the observation area before its purple‐to‐yellow transition, at 4 mL/h the transition occurs in the center of the tubing, and at 8 mL/h the band stabilizes near the tubing‐to‐waste vial exit, partly shifting out of the tubing into the waste vial. These relations hold for all prints, and under a range of flow rates (Figure 5i): in the presence of the mixer (dash‐dotted curve), the standing wave stabilizes ahead of the no‐print case (dotted), which stabilizes ahead of the taper print (solid).

The observed behavior stems from a complex process. Earlier work by Galanics et al. [29], showed that, at perfect mixing, bands form at distinct distances from the entry based only on flow rate, where distance is effectively the equivalence of the time evolution as seen in our CSTR setup (Figure 5c). While this is also true in our fluidic system, we break with the classical flow distance‐to‐time correlation by printing patterns that set the degree of mixing as an additional, programmable control layer. Now, the interplay of fluidic mixing in the context of chemical reaction dynamics and mixing patterns sets the distance at which band formation occurs. The efficient mixer (dash‐dotted) causes the reaction to start earlier, leading to the shortest distance to the purple band. Inversely, the presence of the taper print (solid) discourages mixing, leading to the standing wave appearing further downstream in the tubing. Importantly, the effect is a function of mixing quality, not just of volume as seen by comparing the taper and the mixer, which both occupy similar volumes yet produce very different band positions.

**FIGURE 6:**
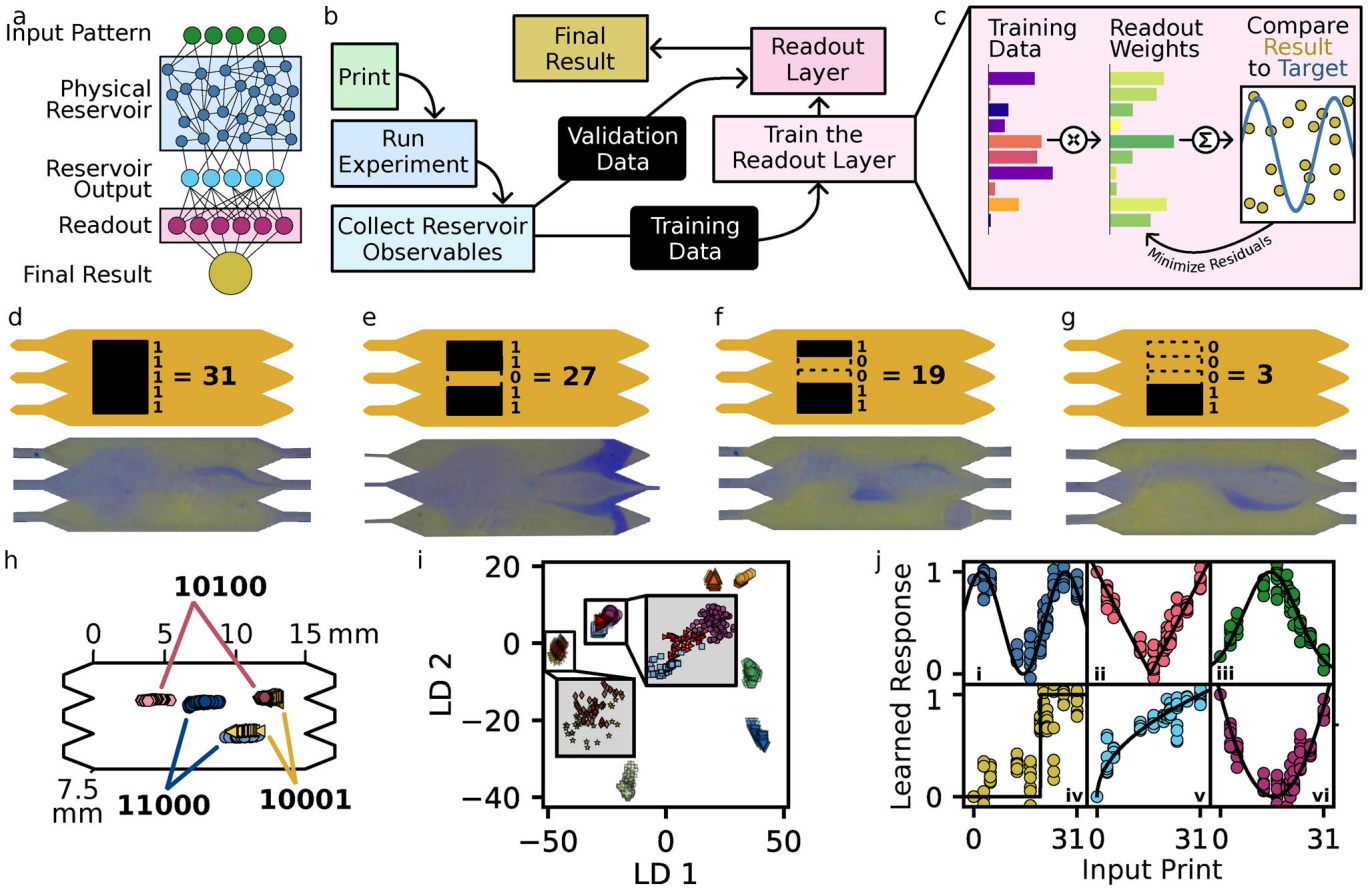
Reservoir computing using the flowing CRN modulated by printed objects. (a) Architecture of a reservoir computer: a physical reservoir converts the input pattern into the reservoir output, which is interpreted by a readout layer to yield the final result. (b) The workflow for the reservoir computer: first, we print the input pattern, the experiment is run, and the resulting reservoir output observables are collected. The collected frames are split up into validation and training data. The training data is used to train the readout layer (detailed in panel c), the validation data is interpreted by the trained readout to yield a final result. (c) The readout works by multiplying the observables of the training output at a single time point by weights and summing the result to yield the final result. To train the readout, the weights are adjusted to minimize the difference between the target value and the results. (d–g) Raw experimental data: Input patterns in the top row. Reservoir output, steady‐state purple‐yellow chemical patterns that develop in the bottom row. (h) Center of mass of the purple regions (with temporal variation, 2 regions per input) observed when printing the 10001 (blue), 11000 (yellow), and 10100 (red) patterns. (i) Result of a linear discriminant analysis (LDA) on all reservoir outputs. Different colors/markers indicate different inputs with temporal variation. (j) After training of the readout weights, the physical reservoir can resolve a sine (i, R^2^ = 0.91), absolute value (ii, R^2^ = 0.91), Gaussian (iii, R^2^ = 0.89), detection (iv, R^2^ = 0.73), square root (v, R^2^ = 0.87), and square (vi, R^2^ = 0.88) function. Details about the learning process and fitting can be found in Method S3.

Since mixing inside the chip is unique to the obstacles printed within, and non‐linear CRNs are exceptionally sensitive to ingredient mixing, we further hypothesized that this combination can be leveraged for in‐chip reservoir computing. Reservoir computing (Figure 6a) requires an input, a reservoir that deterministically transforms and mixes the input non‐linearly, and a resulting measurable reservoir output, which is subsequently interpreted by a trained readout layer. In our system, the reservoir is formed by the coupled dynamics of flow, reaction kinetics, and geometry. Two inert solutions enter the microfluidic chip separately (Figure 5d); upon encountering each other they mix, initiating the CRN. The degree of mixing – that can be changed by adding printed fluid obstacles – and the residence time in a given configuration determine the local reaction outcome that propagates downstream. Each fluid obstacle pattern (the input) alters the mixing state and nonlinearly steers the spatiotemporal CRN dynamics and the spatial CRN output pattern. Characteristic purple regions (high pH) form as a result and are taken as the reservoir output. Through the print–erase cycle of our system, we can easily screen input patterns using the same chip.

To begin with, we lowered the flow rate to 0.25 mL/h to force reactions inside the chip. We next defined 5 adjacent rectangular boxes (1 mm by 3 mm each) inside the chip to generate binary input patterns (Figure 6d–g, Figure S5, Note S4). Each rectangle can be left blank (0), allowing unobstructed flow, or printed (1), forcing the flow around it. There are 2^5^ = 32 possible combinations of printed and blank boxes, each defined by a binary code (00000 … 11111). Figure 6 shows several representative permutations: Figure 6d has all boxes filled (11111), 6e has 1 box unfilled (11011), 6f has two unprinted boxes (10011), etc. When flowing simple dye solutions through the chambers, only simple mixing patterns form, reminiscent of those shown in Figure 3 (see Figure S6). However, for the CRN, much more complex behavior is observed. Purple regions form at hard‐to‐predict places, unique to the particular printed pattern. The system settles into steady‐state patterns (Figure 6d–g), although small variations occur due to the intrinsic dynamics of the system (see Video S4). These purple patterns arise from time‐integrated reaction–transport dynamics, such that the observed states implicitly encode upstream mixing and reaction history. Note that the detailed spatial reaction dynamics do not need to be quantified in reservoir computing, as long as each input settles into quantifiable outputs. This relates, for instance, also to the formose reservoir computer [21], where not every chemical reaction of the formose reaction needs to be quantified, or to, for instance, reservoir computers based on waves [44], where the hydrodynamics leading to patterns do not need to be resolved.

In consequence, for analysis of these patterns, we collected 20 output observables of the system state from each video frame (region size, shape, movement, etc., Method S3 for details). Exemplarily, Figure 6h shows the center of mass of the purple regions that form for different input prints. Slight temporal drift occurs as indicated by symbols of the same color. Based on these observables, we perform a linear discriminant analysis (LDA, Figure 6i) to quantify the separability of reservoir states corresponding to different input patterns. The clear separation of data from different input patterns shows that while the individual output timepoints from the same input are not equal, they are more alike than output timepoints with different inputs.

With the data gathered, we take the final step to use our system as a reservoir computer. Each input pattern is treated as a 5‐bit binary number between 0 and 31 (e.g., 10011: 19). For each input, we keep 10 timepoints for validation and use the rest for training (between 25 and 126 timepoints per input print). Each timepoint contains the 20 reservoir output observables describing the system state. The readout layer is simple: it multiplies the 20 parameters with weights and sums the results to yield a final result. During training, weights are adjusted to make the final result match a specific target function: if the target function is a square root, the target output for input 25 is $\sqrt{25}=5$, and for input 4 it is $\sqrt{4}=2$. The final adjusted weights are our trained readout layer.

We performed training to target six demonstrative non‐linear functions: a sine, the absolute value, a Gaussian, a threshold, a square root, and a square. These functions cannot be approximated by a simple linear process, but require non‐trivial information processing [31]. Next, we feed the validation data to the trained readout layer and plotted the resulting predictions with the target functions (Figure 6j). The reservoir accurately reproduces the six functions, despite experimental noise. This demonstrates that, unlike the binary AND/OR logic gates shown above (Figure 4), computation here is analogue, with continuous‐valued input–output mappings generated by the physical reservoir itself. Crucially, the computational richness does not arise from tuning molecular parameters, but from reconfiguring the physical boundary conditions, demonstrating that adaptable hardware alone can expand the computational state space of a fixed chemical system.

## 3 Discussion

The results presented here demonstrate that the physical geometry of a chemical computing environment can be treated as a dynamic, programmable variable, rather than a static constraint. By utilizing hydrogel structures that can be printed and erased in situ, we have introduced a *soft hardware* control layer that operates independently of the molecular *software* flowing through it. This approach addresses a fundamental limitation in chemical and physical computing, where changes in computational function typically require redesign of the molecular components themselves.

Our switchable DNA logic gates highlight this: Conventionally, changing an operation from AND to OR necessitates redesigning the DNA strands. Here, we achieved this solely by modifying the microfluidic hydrogel topology, which governs how reagents are mixed and routed. This approach suggests a route toward more universal chemical logic systems, in which fixed molecular *software* can be repurposed across multiple computational tasks by modifying only the physical environment. Importantly, this also alleviates long‐standing challenges of orthogonality in large DNA logic systems [10, 45] by allowing a single computing system to have multiple concurrent functions. Concatenation of flow‐through chips could further enhance modularity and computational power.

Beyond digital logic, the manipulation of the CRN illustrates how physical boundaries can profoundly shape spatiotemporal chemical dynamics. Geometry‐dependent standing waves emerge, which would not be addressable in static environments, and which are a direct result of the mixing pattern encoded upstream. This demonstrates that physical structuring provides access to complex chemical states without altering reaction chemistry. In this sense, computation is encoded not only in reaction kinetics, but in the interaction between flow, transport, and boundary conditions.

Leveraging this interplay, we introduce a physical reservoir computer in which printed microfluidic patterns act as inputs that sculpt the nonlinear dynamics of a flowing CRN. The reservoir maps discrete input patterns onto high‐dimensional, continuous‐valued chemical states, from which diverse nonlinear functions can be extracted using a simple linear readout (see Note S4 for a discussion of computational capabilities and limitations). Because only the readout layer is trained, the detailed mechanisms by which the emergent chemical patterns arise (a specific reaction step, transport, mixing, etc.) do not need to be interpreted or even identified; the reservoir functions as a black box whose internal complexity is harnessed without being explicitly understood [21, 22, 44]. Unlike the binary logic gates, computation here is inherently analogue, with continuous input–output mappings generated by the physical reservoir itself. Crucially, the computational richness does not arise from tuning molecular parameters, but from reconfiguring the physical hardware, underscoring that adaptable geometry alone can expand the computational state space of a fixed chemical system.

Our approach is complementary to recent chemical reservoir computing implementations based on intrinsically complex reaction networks, such as the formose reaction [21]. In those systems, computational power arises primarily from the internal chemical network dynamics under time‐varying chemical inputs, while the physical reactor geometry remains fixed. In contrast, our platform introduces a reconfigurable physical structure as an independent computational degree of freedom, enabling the reservoir itself to be reshaped on demand. The present work focuses on static input patterns and readout‐only learning. However, the ability to rewrite microfluidic geometry in situ naturally enables future closed‐loop architectures in which computational performance feeds back to modify the physical reservoir itself. Such hardware‐in‐the‐loop adaptation would blur the boundary between computation, sensing, and material response.

Looking forward, the integration of reconfigurable soft hardware with chemical systems offers a pathway toward truly adaptive matter, in which computation emerges from the co‐design of chemistry, flow, and structure. While the current demonstrations rely on manual pattern updates and operate on minute timescales, integration with automated feedback and responsive materials could enable self‐optimizing chemical computers, smart sensing platforms, and physically embodied information processing in environments inaccessible to conventional electronics. More broadly, our results demonstrate that the physical container of a chemical reaction should not be seen as a passive vessel, but as an active, programmable participant in the computation itself.

## 4 Methods

### 4.1 Reagents

All DNA strands were purchased as HPLC‐purified and used as received (*Biomers GmbH*). Sequences are in Table S1. All other reagents are in Table S2.

### 4.2 Reconfigurable Hydrogel Composition

Reconfigurable hydrogel resin composition: 87.15 wt.% water, 12.25 wt.% acrylamide (AAm), 0.30 wt.% N, N'‐bis(acryloyl)cystamine (BAC), and 0.30 wt.% lithium‐phenyl‐2,4,6‐trimethylbenzoylphosphinate (LAP) (initiator). Resin is used without pre‐treatment or degassing. The resin composition balances print quality and ease of removal. Adding more BAC causes stiffer prints but requires longer reset incubation times. In experiments requiring fluorescence microscopy, ∼0.02 mol% acryloxyethyl thiocarbamoyl Rhodamine was added. After printing, chips are flushed with copious amounts of MilliQ water to remove unreacted resins. Printed structures were reset by incubating prints in a 50 mM aqueous solution of tris(2‐carboxyethyl)phosphine (TCEP) for ca. 20 min. Other reducing agents work similarly (Note S1). Chips were flushed with MilliQ water after dissolution.

### 4.3 Printing

Printing of the reconfigurable hydrogel was done in our printing setup, schematically shown in Figure 2b, consisting of an *Invision Helios* Light Engine (2560 × 1600 resolution, 6 µm/pixel). The light engine can display arbitrary patterns of UV‐light (385 nm) into microfluidic chips and is controlled using custom software [46]. For this resin, we optimized printing to a 5 s, 65 000 µW/cm^2^ pulse.

### 4.4 Microfluidic Setup

#### 4.4.1 Microfluidic Chips

Experiments are performed in custom microfluidic chips. Chips were either made of glass (when optical inspection is required, made to our design by *LightFab GmbH*), or fabricated in‐house by gluing a 1 mm glass coverslip onto a 3D‐printed chip design (printed using an *Asiga MAX X27* DLP printer, *Asiga PlasClear* resin). The internal chip design is identical in both cases, CAD‐files are available with this paper. The total internal volume of the chamber is 90 µL. We note that classic PDMS‐based chips are incompatible with the process (Note S3).

#### 4.4.2 Pumps & Tubing

All microfluidic experiments were driven using *HARVARD apparatus PHD2000 Infusion* syringe pumps, controlled by software developed in‐house for automation of experiments [47]. We used PTFE tubing in all experiments. For experiments involving DNA, we used tubing with a 0.4 mm inner diameter and 0.8 mm outer diameter. In all other experiments, tubing with a 1.59 mm outer diameter and 0.8 mm inner diameter was used.

### 4.5 Microscopy and Imaging

All fluorescence microscopy was performed on a *Nikon SMZ25* stereo microscope. All regular color photographs and videos were taken using *Panasonic DMC G‐70* cameras.

### 4.6 Mixing Flow

In the mixing flow experiments (Figure 3), the dye concentration at entry is 12.5 µm for Sulforhodamine 101 and 20 µm for fluorescein. Fluorescence imaging was performed using standard TR and FITC filter sets. The obtained ratio between the fluorophores was obtained by min‐max normalizing the signal of the individual channels within the chip, before taking the ratio of the resulting normalized intensities. The exit channel profiles of Figure 3c are taken at 0.2 mm before the chip exit, taking the average over a 0.1 mm width.

### 4.7 DNA‐Based Logic Gates

DNA concentrations and molar ratios are constant: 1 × *Inputs*, 1 × *Reporters A & B*, 2 × *Gate*, 2 × *Gate part* and 3 × *Reporter C*, where 1× is equal to 100 nm. Experiments are always performed in TE buffer (pH of 8.0) with 12.5 mm MgCl_2_. Double‐stranded constructs were prepared by mixing strands and annealing by heating to 90°C and cooling to 25 °C at 2.2 °C/min. Double‐stranded DNA molar stoichiometries are given with sequences in Table S1. Experiments were performed at room temperature (RT, 21°C). After the experiments, samples were collected and stored at RT until a set was complete, after which we pipetted 15 µL of each sample (in fourfold) into a Corning black 384‐well plate. Fluorescent measurements were performed on a *Tecan Spark* Plate Reader: FAM with exc. filter 488/20 and em. filter 535/20, Cy5 with exc. filter 633/20 and em. filter 678/20. Bulk experiments were performed directly in the well plate, also at RT, where reagents were added using a *Dispendix I.DOT* liquid handler.

Normalizations in Figure 4 are performed as follows: both FAM and Cy5 signals are normalized such that an intensity of 0 is equal to the unactivated reporters (*Reporters A&B* in the FAM case, *Reporter C* in the Cy5 case), and an intensity of 1 is equal to bulk activation of the reporter.

In the experiments shown in Figure 4, both the in‐flow rate and the out‐flow rate are controlled to ensure the left and right outlet receive an equal amount of material.

### 4.8 pH Oscillator

The aqueous chemical oscillator is based on thiosulfate and hydrogen peroxide, catalyzed by Cu^2+^‐ions as comprehensively characterized by Orban and Epstein [43]. In our experiments, we set the concentrations in the reactions as follows: 50 mm H_2_O_2_, 5 mm Na_2_S_2_O_3_·5H_2_O, 1 mm H_2_SO_4_, 25 µm CuSO_4_, and 5 mm bromocresol purple for visualization. We inject the reaction mixture into the chip in two parts (Figure 5d): the inner channel (red) contains only the H_2_O_2_, while the outer channels (blue) contain all other components. A video of the oscillator in action is provided in Video S1. In the CSTR experiment shown in Figure 5c, we used a 3D‐printed reaction vessel (volume of 700 µL, *Asiga PlasClear* resin, CAD‐design provided with the paper). A 9.0 mL/h flow through the CSTR yields the plotted oscillations. The intensity along the tubing shown in Figure 5g was obtained by taking the relative blue signal compared to the total brightness of the image along the path of the outlet channel. We further performed a Gaussian blur (50 px, ∼1.38 mm) and a rolling mean over 100 px (∼2.75 mm) to smooth the data, and remove regions where dust obscured the data.

## Conflicts of Interest

The authors declare no conflict of interest.

## Supporting information




**Supporting File 1**: adma73669‐sup‐0001‐SuppMat.pdf.

**Supporting File 2**: adma73669‐sup‐0002‐VideoS1.mp4

**Supporting File 3**: adma73669‐sup‐0003‐VideoS2.mp4

**Supporting File 4**: adma73669‐sup‐0004‐VideoS3.mp4.

**Supporting File 5**: adma73669‐sup‐0005‐VideoS4.mp4.

**Supporting File 6**: adma73669‐sup‐0006‐STLs.7z.

## Data Availability

The data that support the findings of this study are available from the corresponding author upon request.
