# Supplementary material for: Soft Hardware, Flowing Software: Reconfigurable Microfluidics for Adaptable Chemical Computation
Source: Adv Mater. 2026 Jun 10;38(39):e73669. doi: 10.1002/adma.73669 (PMC13361266; doi:10.1002/adma.73669)
Supplement: Supplementary file 1 — Supporting File 1: adma73669‐sup‐0001‐SuppMat.pdf. [file ADMA-38-e73669-s006.pdf]

Supplementary Information for

Soft Hardware, Flowing Software: Reconfigurable Microfluidics for Adaptable Chemical Computation

Piet J.M. Swinkels[1], Brigitta Dúzs[1], Oliver Skarsetz[1], Kohei Nishiyama[1], and Andreas Walther[1]

[1] Life-Like Materials and Systems, Department of Chemistry, University of Mainz, Duesbergweg 10-14, 55128 Mainz, Germany.

TABLE OF CONTENTS

Supplementary Figures2

Supplementary Tables5

Supplementary Methods7

Supplementary Method 1: COMSOL Simulations7

Supplementary Method 2: Performing Reservoir Computing7

Supplementary Method 3: Synthesis of LAP8

Supplementary Notes9

Supplementary Note 1: Selection of Reducing Agent9

Supplementary Note 2: The Print-erase Cycle9

Supplementary Note 3: The Reconfigurable Resin and PDMS Chips10

Supplementary Note 4: Computational Capabilities of the Microfluidic Reservoir Computer10

SUPPLEMENTARY FIGURES

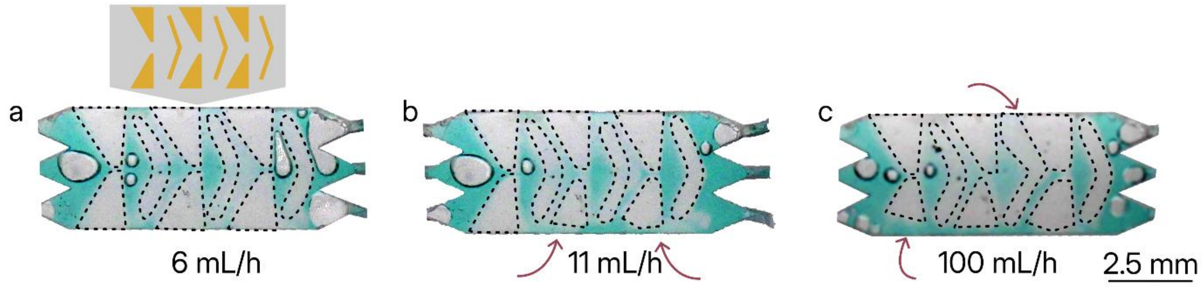

Supplementary Figure 1. Delamination and deformation of hydrogel in the microfluidic chip under high flow rates. The chamber-wedges mixer pattern (inset of a) is printed inside the microfluidic chip. Dyed water (Acid Blue 9) is flown through the chip. We increase the flow rate stepwise from 1 mL/h to 100 mL/h and observe the effect on printed structures. Pictures at a) 6 mL/h, b) 11 mL/h, and c) 100 mL/h are included here, with dotted line guiding the eye to the gel-water interface. At 11 mL/h, the hydrogel delaminates from the vertical wall, indicated by the arrows, but no significant deformation of the gel is visible. At 100 mL/h, the hydrogel has still not delaminated completely, nor has it been broken, but it has deformed significantly. In the light of these results, we advise a maximum flow rate of 6 mL/h.

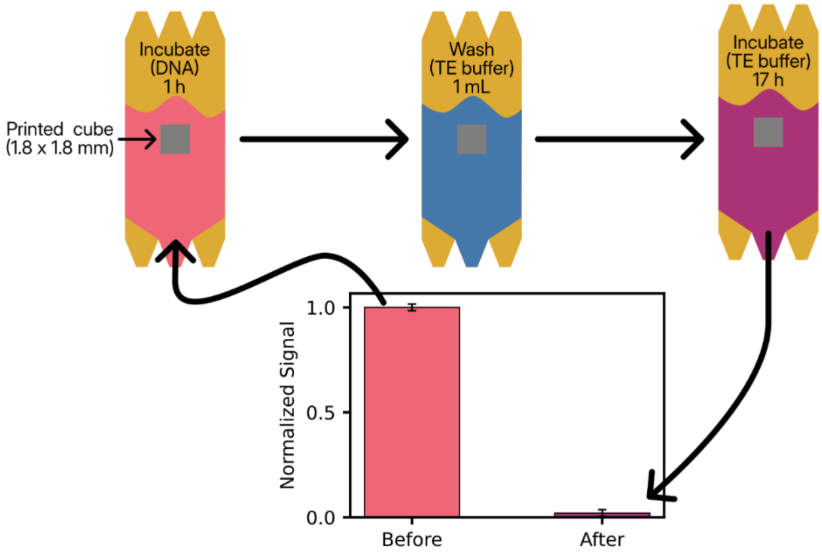

Supplementary Figure 2. DNA does not adhere to or absorb into printed structures. A 1.8 x 1.8 mm cube is printed into the microfluidic chip with the reconfigurable resin, as described in the Methods of the main text. We then inject a 300 nM solution of FAM-functionalized DNA (*Reporter B (forward)*, see Supplementary Table 1). We let the DNA incubate for 1 hour at room temperature (RT). The chip is then flushed with 1 mL of TE buffer, enough to remove any traces of DNA solution, leaving only potential DNA adhered or absorbed to the printed cube. We then inject fresh TE buffer, and incubate it overnight at RT. The injected TE buffer is recovered, and its fluorescence measured in a plate reader, together with the initial DNA solution. The incubated buffer shows only background fluorescence, indicating that the amount of DNA accumulating is minimal. Error bar indicates the standard deviation of 3 repeats. The normalization is set such that TE buffer is 0 and the DNA solution is 1.

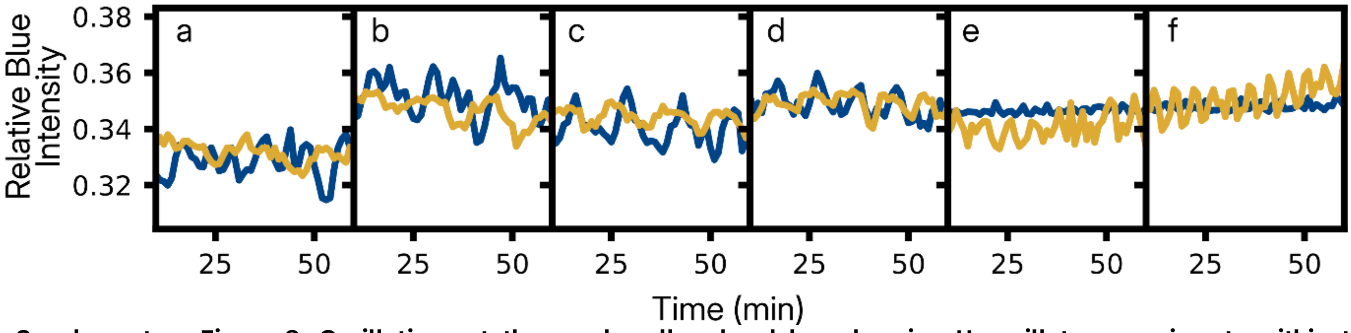

Supplementary Figure 3. Oscillations at the purple-yellow band boundary in pH oscillator experiments within the downstream tubing (see also Supporting Video 2 and 3)**.** Relative blue intensity measured on the edge of the blue band region (see Figure 5g of the main text) over the course of an experiment. Yellow traces are from chips without a printed structure, blue traces from chips with a chamber-wedge mixer. The panels show different flowrates: a) 1.92 mL/h, b) 2.40 mL/h, c) 2.88 mL/h, d) 3.84 mL/h, e) 6.00 mL/h, f) 8.00 mL/h.

The time-resolved signal at the purple-to-yellow transition shows small scale oscillations (see Supplementary Videos 2 & 3) which are not random noise but structured. Oscillations appear at every flow rate, though their amplitude and period vary; the period ranges from 5 to 15 min, too slow to be pump artifacts, which are on the second scale[1]. This suggests these oscillations are not an artifact of our methods, but an inherent feature of the system.

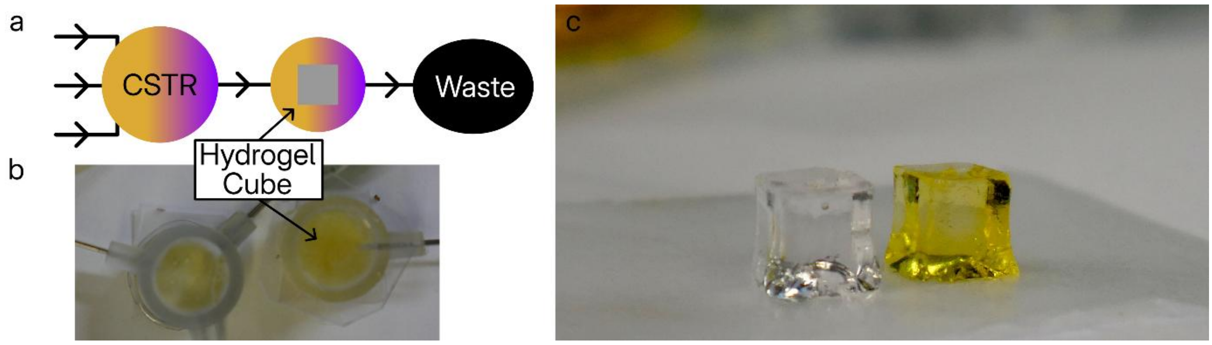

Supplementary Figure 4. Hydrogel stability in the pH-oscillator. The pH oscillator is run macroscopically in a continuously stirred tank reactor (CSTR), like the experiment in Figure 5a-c of the main text, with the addition of a second reaction chamber following the CSTR. Other parameters (concentrations, flow rate, …) are kept the same as the parameters in the main text. In this secondary reaction chamber, we place a 5x5x5 mm hydrogel cube. When the CRN oscillates in the CSTR, it also oscillates inside this chamber. We let the reaction run for 1 hour and recover the hydrogel cube. a) A sketch of reactor design. b) A picture of the CSTR and the chamber containing the hydrogel cube during the reaction. c) An otherwise identical cube is soaked in deionized water for the duration of the experiment and compared to the hydrogel cube from the reactor. The dimensions of the two cubes are identical, and no degradation of the cube is observed. The only obvious difference is the color, caused by the diffusion of the bromocresol purple into the hydrogel,

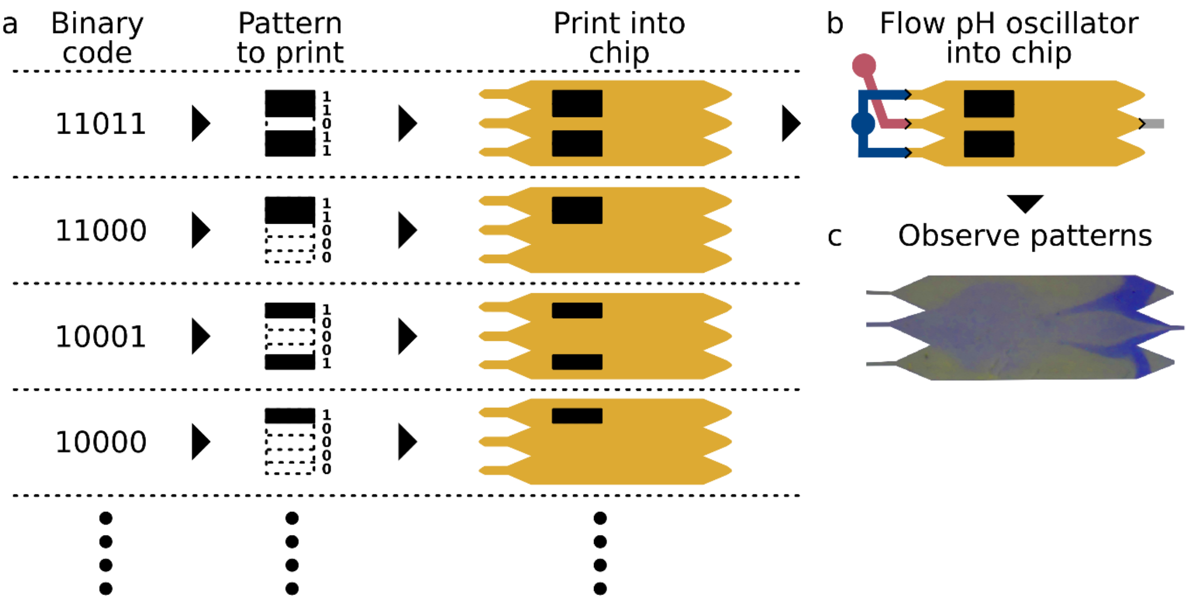

Supplementary Figure 5: Encoding binary code as a print pattern and observing its effects on the pH oscillator. a) A 5-digit binary code consisting of 1s and 0s is converted into a printable pattern consisting of 5 rectangular boxes. If the digit is 0, nothing is printed, if the digit is 1, a box is printed. Different binary codes thus result in similar but discrete printed patterns. There are 32 possible patterns. b) After printing a pattern, the pH oscillator flows into the chip. The flow in the chip is sculpted by the printed object. c) The sculpted flow and mixing lead to complex reaction–transport dynamics, resulting in the observed purple patterns,

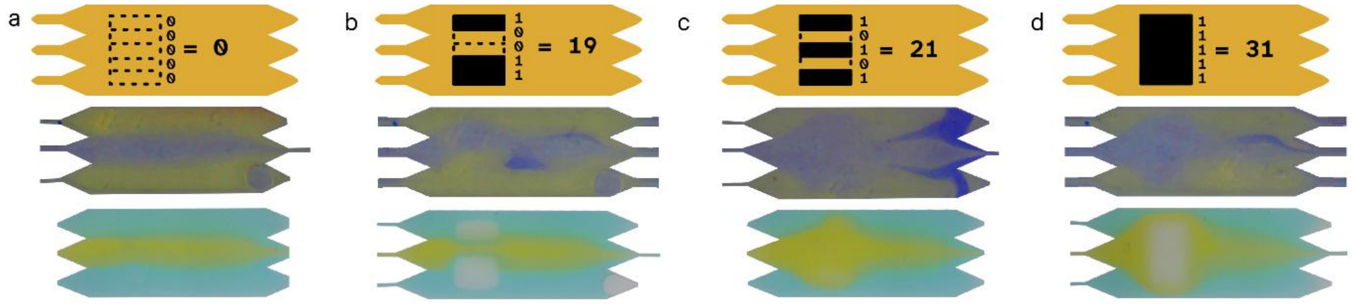

Supplementary Figure 6. Chemical dynamics in a CRN are needed to form complex distinguishable patterns for reservoir computing. A comparison of mixing as a result of printed objects between the CRN (pH oscillator) discussed in the main text and passive dyes, all performed at 0.25 mL/h.

The **top row** shows the **printed objects** for each of panel a to d.

The **middle row** shows the steady-state of the CRN in flow when performed as discussed in the main text, Figure 6.

The **bottom row** shows the result of flowing dyed water, outer inlets (blue) are dyed with Acid Blue 9, central inlet (yellow) is dyed with tartrazine using the same microfluidic setup.

Uniqueness of patterns and features is much greater for CRNs.

SUPPLEMENTARY TABLES

Supplementary Table 1. DNA strands used in this work.

Name	Sequence (5' to 3')	5'-modification	3'-modification	Double-stranded Molar Ratio
Input A	tac tca tca cca ctt aac cta c			
Input B	cta atc aac aca cct act atc a			
Reporter A (forward)	gta ggt taa gtg gtg atg agt a	6-FAM		1.0
Reporter A (reverse)	cac cac tta acc tac		BMN-Q1	1.1
Reporter B (forward)	tga tag tag gtg tgt tga tta g	6-FAM		1.0
Reporter B (reverse)	cta atc aac aca cct		BMN-Q535	1.1
Gate (forward, "gate part")	cac cac tta acc tac taa ctc taa tca aca cac ct			1.0
Gate (reverse)	tga tag tag gtg tgt tga tta gag tta gta ggt taa gtg gtg atg agt a			1.1
Reporter C (forward)	agg tgt gtt gat tag agt ta	Cyanine 5		1.0
Reporter C (reverse)	cta atc aac aca cct		BBQ-650	1.5

Supplementary Table 2. Reagents used in this work.

Name	Acronym	Supplier	Purity/Grade
Acrylamide	AAm	TCI	98 %
N,N'-bis(acryloyl)cystamine	BAC	Fisher Scientific	98 %
Lithium-Phenyl-2,4,6-trimethylbenzoylphosphinate	LAP	Synthesized, see Supplementary Methods 1	
Ethyl mesitylcarbonyl (phenyl) phosphinate		Carbosynth	
2-butanone		Carl Roth	
Lithium Bromide		Alfa Aesar	anhydrous
Acryloxyethyl thiocarbamoyl Rhodamine B		Polysciences	
Tris(2-carboxyethyl)phosphine hydrochloride	TCEP	BLDpharm	
Fluorescein (sodium salt)		Sigma-Aldrich	
Rhodamine B		Merck Millipore	
Sulforhodamine 101		TCI	>95 %
Magnesiumchloride hexahydrate		VWR	
TE buffer		Thermo-Fisher	pH 8.0
Bromocresol purple	BCP	Alfa Aesar	
Hydrogen peroxide		Acros Organics	35 %
Sodium thiosulfate pentahydrate		VWR	99.5 %
Sulfuric Acid		VWR	95 %
Copper(II) sulfate pentahydrate		Sigma-Aldrich	98 %
Tartrazine		BLDpharm	85%
Acid Blue 9		TCI	>99%
Dithiothreitol		TCI	98%
Cysteine		Sigma-Aldrich	

Supplementary Table 3. The 15 main contributing dimensions to the LDA-axis.

Dimension	Relating to Biggest or Smallest region	LD1 magnitude	LD2 magnitude
y-location	Biggest	28.487	6.093
y-velocity	Smallest	-26.587	16.463
Number of purple regions	-	23.667	-49.686
x-location	Smallest	-21.325	45.751
Standard deviation of y-velocity	Smallest	-14.782	10.179
Eccentricity	Smallest	11.384	-33.735
Standard deviation of x-velocity	Smallest	9.628	-1.792
x-velocity	Smallest	5.676	-11.534
y-location	Smallest	4.391	1.527
Orientation	Smallest	3.799	1.315
Standard Deviation of y-location	Smallest	2.638	31.860
Standard Deviation of x-location	Smallest	2.540	-14.236
Orientation	Biggest	-2.181	0.330
Total Area	Smallest	-2.084	-4.819
Eccentricity	Biggest	1.974	1.421

SUPPLEMENTARY METHODS

Supplementary Method 1: COMSOL Simulations

Simulations are performed on COMSOL Multiphysics 6.3, with CFD and microfluidics modules. The simulated geometry is defined to match our microfluidic chips after printing.

The flow in the chip is assumed to be laminar, with walls having the 'no slip' boundary condition. Hydrogel objects in the channel are modelled as static walls which do not interact with flow or diffusion. Flow inlets have a fixed mass flow rate equal to the experimentally set flow rates. Outlets are given a 1 atm static pressure, and backflow is suppressed.

Transport of diluted species in the channel is modelled by assuming a diffusion coefficient of 4.0E-10 m^2s^{-1}, consistent with small fluorescent molecules like Rhodamine B. The transport of the diluted species is coupled to the laminar flow using the *Reacting Flow* multiphysics interface.

Raw COMSOL files are available upon request.

Supplementary Method 2: Performing Reservoir Computing

Extracting Data

From videos of the purple pulses, such as Supplementary Video 4, we first extract as much quantifiable data as possible. For each printed object, we capture a >60 minutes long video, with a frame taken every 30 seconds. We discard the start of the movie, where our system has not yet reached a steady state (the first 5-10 minutes). The video is then clipped to only contain the chip area (and not the surrounding area), aligned, and scaled. We then threshold the video, so only the purple areas remain.

We did not perform experiments on all 32 possible prints. To make our data go further, we used data from non-symmetric prints (e.g., 10000, 11000) and simply mirrored it to yield results for their mirrored counterparts (e.g., 00001, 00011). Since our system is fully symmetric around the central axis, this has no influence on output.

We then extract 20 different parameters from the resulting thresholded data. Since most (but not all) printed structures lead to two purple regions, we extract most parameters (dimensions) for both the smallest and the biggest detected area. The parameters extracted are:

- The number of distinct purple regions.
- x- and y-location of the region, smoothed with a 5-frame (=2.5 min) rolling average.
- Standard deviation of the x- and y-location of the region, rolled over 5 frames.
- The x- and y-velocity of the region smoothed with a 5-frame rolling average.
- The standard deviation of the x- and y-velocity of the region rolled over 5 frames.
- The area of the region.
- The area of the bounding box of the region.
- The eccentricity of the region shape.
- The orientation of the region.

More parameters (e.g., the displacement to path length ratio) could be extracted from the results. However, LDA analysis and fitting reveals that more parameters do not improve results further. We further note that the smaller of the two purple regions that typically form seems to be the more important for distinguishing different input prints. In Supplementary Table 3, we show the 15 main contributing dimensions and their relative magnitude to both LDA-axis of Figure 6i of the main text. Of the 15 properties listed, 11 relate to the smaller of the purple regions. This may indicate that the smaller region, if it is present, has the most morphological differences, and is more caused by the print, while the primary bigger region has its location and dynamics set more by the basic chip design

The parameters listed are easily extracted using the Python scikit-learn package. Missing data (e.g., if there is only one purple region) is given a placeholder value of -10, which does not naturally appear in the datasets – this is necessary because missing data is not handled gracefully by the linear regression (readout) step.

Training

Now that we have extracted these parameters, we have an input (e.g. 10011: 19) associated with quantitative output observables. We take all timepoints we have for each input and split it into *training* data and *validation* data. For each input, we keep 10 timepoints for the validation and use the rest for training (between 25 and 126 points per input print). Data is assigned to the two categories randomly to prevent bias. We now select a function to train for. In Figure 6j of the main text, we select six non-linear functions:

i. A sine: $f(x) = 0.5 \cdot sin\left(\frac{1}{4} \cdot x + 1\right) + 0.5$
ii. An absolute value: $g(x) = |x - 16|/16$
iii. A Gaussian: $h(x) = exp\,(-\frac{(x-15)^2}{256})$
iv. A detector: $k(x) = \begin{cases} 0 x < 20 \\ 1 x \geq 20 \end{cases}$
v. A square root: $l(x) = \frac{\sqrt{x}}{\sqrt{31}}$
vi. A square: $m(x) = \frac{x^2}{31^2}$

Where x is the input (e.g. when printing 10011, x equals 19).

The training consists of a linear regression fit of the training data for each of the above functions. Each print is weighted equally: the number of timepoints in a particular experiment does not influence the fit. After fitting, we feed the validation data to the model and plot the result in Figure 6j of the main text.

The full procedure is carried out in Python using the scikit-learn module, which is open source. The code is available upon request.

Supplementary Method 3: Synthesis of LAP

Synthesis of LAP was performed according to previously reported procedure[2]. Ethyl mesitylcarbonyl (phenyl) phosphinate (5.69 g, 18.0 mmol) was dissolved in 2-butanone (100 mL). Lithium bromide (6.25 g, 72.0 mmol) was added, and the mixture was heated to 50 °C for 15 min, allowed to cool to room temperature, and left to stand overnight. The crystallized product was recovered by filtration, washed with ice-cold 2-butanone, and dried in vacuo to yield LAP as a fine white powder in quantitative yield.

SUPPLEMENTARY NOTES

Supplementary Note 1: Selection of Reducing Agent

In the main text, we always perform the cleaving of BAC cross-links using a 50 mM of TCEP , which has a redox potential of -0.29 V. TCEP is highly efficient and removes crosslinks quick and without much trouble. We also tested two other agents: we prepared aqueous solutions of cysteine (redox potential of -0.22 V), dithiothreitol (redox potential of -0.33 V), all diluted to 50 mM (a clear over-stoichiometric amount compared to cross-linker concentration), and add a small (150 μL) cube of reconfigurable hydrogel to the solution (see Methods of main text for composition). All cubes dissolve fully after approx. 60 minutes, all at similar rates. Since cysteine leaves a white precipitate, and dithiothreitol has a strong smell, we use TCEP in all our experiments.

Supplementary Note 2: The Print-erase Cycle

In our system, the print-and-erase cycle occurs entirely in-flow:

1. First flow in monomers/ink,
2. print,
3. flush with water,
4. perform an experiment, and
5. reset the chip with TCEP to erase the printed patterns.

Printing takes in the order of seconds; the experiment takes between 10's of minutes and hours (see main text at the relevant sections), and the erasure time is strongly dependent on the printed structure. Taking the mixers in Figure 3b of the main text as a typical example, erasing the structure in *iv* takes in the order ca. 25 minutes, while the pillars in *ii* take only ca. 10 minutes to erase. The difference is due to the larger relative area exposed to the TCEP solution. The total time of the cycle is thus just under 1 hour, depending on print and experiment.

The breakdown rate is determined by two processes: the disulfide reduction rate, and the diffusion of the reducing agent into the hydrogel. Both are fundamental to the reaction and cannot be circumvented. In our system, diffusion is the limiting step; adding more TCEP does not make dissolution faster. Therefore, we see three routes to speeding up this process. (1) Downscaling: small objects have both fewer disulfide bonds and are faster to diffuse into. Downscaling comes with its own sets of issues, like printing resolution, handling difficulty, etc., but we are confident such issues can be solved with time. (2) Introducing porosity: pores in the hydrogel make diffusion easier. Earlier work in our lab shows this is relatively easy to achieve in the printing setup[3]. (3) A smaller reducing agent: hydride salt or other small reducing agents would diffuse into gels more easily, although this obviously comes at the cost of using very hard to handle materials. More elaborate, reversible photochemistry might yet be a complete alternative, but this would require a multicolor printer.

The hydrogel sticks to the glass chip non-specifically; no specific surface treatment is necessary. Between uses, we flush copious amounts of Milli-Q water through the chip, and this has been enough for all re-uses of the glass chips to date. This non-specific adhesion is very convenient, since any surface treatment is likely to degrade over time/with usage. We hypothesize that the non-specific adhesion arises from chemical adhesion and mechanical grip. Chemical adhesion can take place through hydrogen bonding between the polyacrylamide groups of the polymers and silanol groups on the glass. Mechanical grip arises from slight post-print swelling that presses the gel against the channel walls.

As a direct consequence of this, there is no clear upper limit to the number of print-erase cycles we can perform in a chip.

Supplementary Note 3: The Reconfigurable Resin and PDMS Chips

We first tried printing the reconfigurable resin inside PDMS-glass chips – the classic prototyping chip, easy to fabricate in-house. We observe that when printing in such chips, a hydrogel layer formed on the glass but never reached the PDMS ceiling, likely because oxygen dissolved in PDMS quenches radical polymerization near the surface[4]. However, this inhibition hit our resin harder than other systems described in literature using more conventional cross-linkers like *N,N'*-methylenebisacrylamide. Microfluidic control of flows was hard to achieve due to leakage over the printed obstacles. Interestingly, bulk polymerization still worked (see e.g., Figure 1b of the main text). Degassing before experiments had no discernible influence. Rather than optimizing, we simply switched chip material, see Methods section of the main text. The cross-linker may be unusually oxygen-sensitive, though we offer no specific acting mechanism here. Consider this a caution to anyone tempted to try our system with PDMS chips.

Supplementary Note 4: Computational Capabilities of the Microfluidic Reservoir Computer

Existing implementations of chemical reservoir computing (RC) on continuous-flow CRNs have relied on fixed microfluidic geometries with time-correlated inputs[5,6]. By introducing photo-printable and chemically erasable hydrogel obstacles, our system adds a spatial, programmable input dimension that is absent from those platforms. The advance reported here is therefore reconfigurability and input diversity. The same chip can be adapted to different task requirements by altering the flow geometry in situ, enabling the physical reservoir dynamics themselves to be reprogrammed without fabricating new devices.

There is no clear relationship that couples the number of reactions, compounds, or other parameter of a physical reservoir to a "computation power". Nonetheless, there are some general tendencies: more complexity, meaning more reactions, steps, interactions, in the reservoir generally result in more raw computational capability of the reservoir computer[7].

In this work, we use a 5-bit input consisting of 3x1 mm boxes, which is a pragmatic choice as a reasonable starting point. The reservoir would in principle take any print as input, hence, the 5-bit binary pattern is not a fundamental feature of the system. Indeed, the 5-bit system was specifically chosen by us for two reasons: firstly, it is easy to print reproducibly, and secondly, the resulting output patterns are easy to distinguish (meaning, the point clouds in Figure 6i of the manuscript do not overlap). Different bit shapes, sizes, or encodings would likely function similarly, provided they meet these reproducibility and distinguishability constraints for the outputs. Using more complex input patterns would unlock a richer variety of output patterns. Thinking further, as the differences between inputs become subtler, the differences between output states necessarily shrink. Once the point clouds in Figure 6i begin to overlap, the states can no longer be resolved experimentally, regardless of any theoretical complexity the reservoir might possess. The 5-bit system therefore represents a balance between input complexity and reliable readout. The simple yellow-to-purple transition observed here conceals considerable dynamical details in occurring chemical dynamics. Directly probing those states (e.g., via spatially resolved spectroscopy or additional reporter chemistries) could in future allow more complex print designs to be exploited without sacrificing distinguishability, because, next to macroscopic color, different chemical details could be resolved.

Finally, it is worth noting that a particular physical reservoir could be well suited for a particular problem (so generating a specific function in our case), but not for another[7,8]. The processes occurring in a reservoir may just not map well onto a specific problem, and it is impossible to know why without deep understanding of the internal dynamics of the reservoir. The differences in fit quality we observe in Figure 6j of the main text are therefore not easy to pin on a specific feature of our system. In this context, it is interesting to consider how our system could be designed to be more suited to solve a specific class of problems. In the extreme, we return to more "regular" calculations that only solve one specific problem.

Supplementary References

(1) Leuthner, M.; Hayden, O. Grease the Gears: How Lubrication of Syringe Pumps Impacts Microfluidic Flow Precision. *Lab. Chip* **2024**, *24* (1), 56–62. https://doi.org/10.1039/D3LC00698K.
(2) Fairbanks, B. D.; Schwartz, M. P.; Bowman, C. N.; Anseth, K. S. Photoinitiated Polymerization of PEG-Diacrylate with Lithium Phenyl-2,4,6-Trimethylbenzoylphosphinate: Polymerization Rate and

Cytocompatibility. *Biomaterials* **2009**, *30* (35), 6702–6707. https://doi.org/10.1016/j.biomaterials.2009.08.055.
(3) Skarsetz, O.; Swinkels, P. J. M.; Figueiredo da Silva, J.; Vozzolo, G.; Masukawa, M.; Fusi, G.; Dúzs, B.; Lassiat, Y.; Drees, C.; Slesarenko, V.; Walther, A. Soft Robotic Engines with Non-Reciprocal Motion by Physical Intelligence. *Adv. Mater.* **2025**, e11630. https://doi.org/10.1002/adma.202511630.
(4) Dendukuri, D.; Panda, P.; Haghgooie, R.; Kim, J. M.; Hatton, T. A.; Doyle, P. S. Modeling of Oxygen-Inhibited Free Radical Photopolymerization in a PDMS Microfluidic Device. *Macromolecules* **2008**, *41* (22), 8547–8556. https://doi.org/10.1021/ma801219w.
(5) Baltussen, M. G.; de Jong, T. J.; Duez, Q.; Robinson, W. E.; Huck, W. T. S. Chemical Reservoir Computation in a Self-Organizing Reaction Network. *Nature* **2024**, *631* (8021), 549–555. https://doi.org/10.1038/s41586-024-07567-x.
(6) Ghosh, S.; Baltussen, M. G.; Knox, A. C.; Haije, R.; Duez, Q.; Tsitsimeli, A. T.; Chak, M. H.; Beves, J. E.; Huck, W. T. S. A Recursive Enzymatic Competition Network Capable of Multitask Molecular Information Processing. *Nat. Chem.* **2025**, 1–7. https://doi.org/10.1038/s41557-025-01981-y.
(7) Lukoševičius, M. A Practical Guide to Applying Echo State Networks. In *Neural Networks: Tricks of the Trade: Second Edition*; Montavon, G., Orr, G. B., Müller, K.-R., Eds.; Springer: Berlin, Heidelberg, 2012; pp 659–686. https://doi.org/10.1007/978-3-642-35289-8_36.
(8) Cucchi, M.; Abreu, S.; Ciccone, G.; Brunner, D.; Kleemann, H. Hands-on Reservoir Computing: A Tutorial for Practical Implementation. *Neuromorphic Comput. Eng.* **2022**, *2* (3), 032002. https://doi.org/10.1088/2634-4386/ac7db7.
